# New and Old Mechanisms Associated with Hypertension in the Elderly

**DOI:** 10.1155/2012/150107

**Published:** 2011-10-20

**Authors:** Petra J. Mateos-Cáceres, Jose J. Zamorano-León, Pablo Rodríguez-Sierra, Carlos Macaya, Antonio J. López-Farré

**Affiliations:** Cardiovascular Research Unit, Cardiology Department, Hospital Clínico San Carlos, Madrid 28040, Spain

## Abstract

Hypertension is a widely prevalent and important risk factor for cardiovascular diseases that increase with aging. The hallmark of hypertension in the elderly is increased vascular dysfunction. However, the molecular mechanisms by which increased blood pressure leads to vascular injury and impaired endothelial function are not well defined. In the present paper, we will analyze several mechanisms described in the scientific literature involved in hypertension in the elderly as endothelial dysfunction, increased oxygen delivery to tissues, inflammation, cellular apoptosis, and increased concentration of active metabolites. Also, we will focus on new molecular mechanisms involved in hypertension such as telomeres shortening, progenitor cells, circulating microparticles, and epigenetic factors that have appeared as possible causes of hypertension in the elderly. These molecular mechanisms may elucidate different origin for hypertension in the elderly and provide us with new targets for hypertension treatment.

## 1. Introduction

The elderly, considered as individuals 65 years of age and older, represents the most rapidly growing segment of the population. Age is a powerful risk factor for hypertension, death, and cardiovascular death [1]. In this regard, high blood pressure in the elderly confers a three- to fourfold increase in risk for cardiovascular disease, compared to younger individuals [2]. New guidelines have tried to provide evidence-based treatment algorithms in which control of hypertension is just one aspect of general risk factor control, with the aim of decreasing the total risk. According to the World Health Organization, hypertension is the commonest cause of preventable death in developed countries, and it is increasingly significant in developing countries. Particularly, it has been described that hypertension affects more than one half of those aged 65 and older, and its prevalence continues being increased with age. The incidence of hypertension in the elderly population, over age 60–65 years, is very high with prevalence as high as 60% to 80%. It is estimated that two out of three individuals over 75 years of age suffer hypertension [3].

Large number of studies has revealed that patterns of hypertension change with age. In this regard, systolic blood pressure increases, while diastolic blood pressure decreases after the age of 60. These different patterns indicate us diverse etiologic and hemodynamic mechanisms for hypertension in the elderly population [4]. Several mechanisms involved in hypertension in the elderly have been described in the scientific literature as endothelial dysfunction, increased oxygen delivery to tissues, increased concentration of active metabolites or increased myogenic constriction. Recently, new molecular mechanisms involved in hypertension such as telomeres shortening and endothelial progenitors cells have appeared as possible causes of hypertension in the elderly. These molecular mechanisms may elucidate different origin for hypertension in the elderly and provide us with new targets for hypertension treatment. This paper will be focused in the management and old and new molecular mechanisms associated with hypertension in the elderly.

## 2. Management of Hypertension in the Elderly

Due to the advances in the treatment of hypertension, the definition of isolated systolic hypertension has been changed from a blood pressure level ≥160/<90 to ≥140/<90 mmHg. Initially, the focus of hypertension studies was only on diastolic blood pressure. Later, multiple hypertension trials demonstrated that systolic blood pressure (SBP) levels were concomitantly lowered with diastolic blood pressure and that SBP was more closely associated with improvements in outcome than diastolic blood pressure, providing more relevance to SBP.

High blood pressure, and in particular, isolated systolic hypertension (ISH), has been in the elderly, as recently as two decades, ignored as a cardiovascular risk factor [5]. In 1985, the European Working Party on High Blood Pressure in the Elderly (EWPHE), provided the first evidences about the benefits of the therapeutic intervention in the elderly hypertensive patients [6]. However, it was not until the 1990′s when it really took into consideration the fact of treating hypertension based solely on systolic pressure. In 1991, the Systolic Hypertension in the Elderly Program (SHEP) demonstrated in 4736 older individuals with SBP levels >160 mmHg and diastolic blood pressure levels <90 mmHg, randomized to treatment with thiazide-type diuretic-based regimen versus placebo, greater reductions in blood pressure in the treated group and reductions in the primary end point by 36%, heart failure by 49%, and coronary events by 27% [7].

In 1997, the Systolic Hypertension in Europe Study (SYS-TEUR) corroborated the results obtained in the SHEP study. In the SYS-TEUR study a group of aging patients were randomized to the dihydropyridine calcium channel blocker nitrendipine or placebo. In this study, blood pressure was not reduced as effective as in SHEP. However, the benefits obtained were higher, showing a significant reduction in stroke by 42% [8]. 

The Hypertension in the Very Elderly Trial (HYVET) is other remarkable trial of 3854 patients over 80 years of age, who received a combination of indapamide and perindopril. The study was stopped prematurely due to the conclusive results showing that people who received effective antihypertensive treatment were at 74% less risk of developing congestive heart failure and 20% or more at less risk of developing a stroke or dying either from cardiac complications or any other causes of death. These results provided the clinical evidence supporting the most recent guidelines for the treatment of hypertension [9].

## 3. Main Molecular Mechanisms Associated with Hypertension and Aging

### 3.1. Vascular Aging: Endothelial Dysfunction

Arterial wall is constituted for three layers: intima, media, and adventitia. Media and especially intima layers are where major alterations occur with age. Intima layer is based in a layer of endothelial cells on a subendothelial space that is separated from media layer due to elastic fibers. Media layer is formed by smooth muscle cells connected by extracellular matrix, muscle cells are also responsible for releasing of extracellular matrix components such as collagen and elastin. In advancing age, lipids are internalized into elastin fibers, and they attract calcium ions that provoke loss of elasticity and degradation of elastin fibers due to elastases.

As Dr. Osler postulated, “A man is so old as his arteries”. In this regard, recent studies have concluded that structure and function of arteries change throughout the lifetime of humans [10, 11]. So, it has been demonstrated that in humans central elastic arteries as well as arterial wall dilate with advancing age. In addition, a large number of studies using animal models also found an age-associated restructuring of the central arteries of rats, rabbits, and primates [12, 13]. 

Endothelial cells play a central role in regulating several arteries properties, such as releasing of nitric oxide (NO), vascular tone, permeability, and, therefore, in the arterial pressure. Nitric oxide is a multifunctional molecule with an important role in the relationship between the cells that compose the microvascular environment. Perhaps, the most important effect of NO is its vasodilating property [14]. NO provokes vasodilation by stimulating soluble guanylate cyclase in the vascular smooth muscle cells [15]. Therefore, alterations in the expression of NO synthase (NOS) isoforms or its functionality has been widely associated with hypertension.

**Figure 1:**
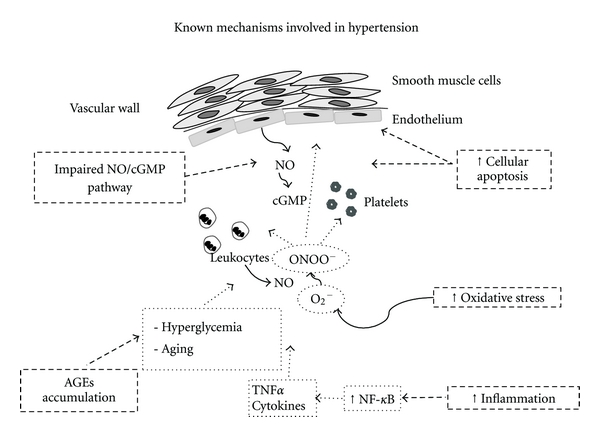
Known mechanisms involved in hypertension in the elderly population. Impairment of the NO/cGMP system, increased cellular apoptosis, and increased concentration of active metabolites such as advanced glycation end products (AGEs) together with an enhanced oxidative an inflammatory state are well-known mechanisms that contributes to the development of hypertension in the elderly population.

Age alters the amount, arrangement, and structural integrity of the endothelial cells cytoskeleton, which affects the mobility, migration, proliferation, and structural integrity of endothelial cells [16, 17]. At molecular level, numerous studies have associated the age advancing with decreased EC capacity for replication and increasing apoptosis, proinflammatory status and reduced production, and/or bioavailability of NO (Figure 1).

### 3.2. Alterations in the Production and/or Bioavailability of Nitric Oxide

Several vascular disorders, including a diminished endothelium-dependent relaxation, have been demonstrated in aging humans and even in experimental animals [18, 19]. Different hypothesis have been raised to explain the reduction of the endothelium-dependent vasodilatation described in the aging humans and animals: a decreased number of vasodilator receptors in the endothelium [20], a diminished capability to generate NO by the endothelium [19], and a reduction in guanylate cyclase activity in vascular smooth muscle cells [21]. In this light of evidences, a study performed by Cernadas et al. revealed new evidences about the function of the NO-dependent vasorelaxing mechanisms in aging rats [22]. They found in aging animals a reduced vasorelaxing response to acetylcholine, an endothelium NO-dependent vasodilator. However, paradoxically, blood vessels from aging rats showed a marked capacity to produce NO through the presence of the inducible NOS (iNOS) isoform. This finding highlights the importance of the presence iNOS isoform in aging, because at sites of endothelial damage, the locally released cytokines, such as tumour necrosis alpha (TNF*α*), could potentially stimulate iNOS expressed in the vascular wall. In this sense, continuous generation of NO by iNOS has been associated with the impairment of the NO system in endothelial cells which has been speculated to protect the vascular wall from excessive amounts of NO. The NO released by the iNOS activity could decrease the eNOS activity, thus favoring the impaired endothelium-dependent vasorelaxation. Indeed, NO has been also described as a cytotoxic molecule for endothelium, inhibiting their growth [23]. In addition, it is well known that free radicals, which increase in aging, inactivate NO by interacting with high concentrations of superoxide anion and resulting in enhanced peroxynitrite formation. Therefore, iNOS may have an important role in the maintenance of vascular tone with increasing age.

Endothelial dysfunction occurs early in several cardiovascular disorders including atherosclerosis, diabetes, and hypertension. Hypertensive subjects exhibit endothelial dysfunction, indicating that endothelial dysfunction precedes the development of clinical hypertension. Systolic hypertension is the dominant form of hypertension in older individuals [24]. Arterial stiffness, determined by structural properties of the blood vessel wall and by smooth muscle tone, is one of the most important factors underlying the increase in systolic pressure. In this regard, longitudinal studies in humans have shown that arterial stiffness is an independent predictor of the rise in systolic blood pressure and of incident hypertension [25]. All these experimental evidences reveal the stretch linkage between aging and hypertension. Recently, a brilliant article performed by the American Society of Hypertension Writing Group [26] recognized the pivotal role of vascular aging in the continuum of cardiovascular risk leading to hypertension.

### 3.3. Endothelial Replication and Apoptosis

Human aging is accompanied by a degeneration of various tissues, which lose part of their physiological functions. Apoptotic cell death plays an important role during aging of various tissues in vivo. Tissue damage due to age-dependent apoptosis has been documented in experimental animals for the brain [27], the inner ear [28], among others. It has been also described that the regulation of programmed cell death plays an important role for the ageing process in vivo. In this regard, atherosclerosis, a major age-related disease of humans, is accompanied by a degeneration of vascular endothelial cells and vascular muscle cells due to programmed cell death or apoptosis [29]. However, the mechanisms leading to age-related apoptosis in an endothelial cell remains to be clarified. In this regard, Warner et al. demonstrated that human endothelial cells followed a different senescence program from the program displayed by human fibroblasts [30]. They found that endothelial cells showed an age-related increase in programmed cell death due to DNA rereplication in the absence of mitosis with G1 arrest, leading in the accumulation of “N” DNA content >4 in endothelial cells. In this light of evidences, Asai et al. also postulated a potential mechanism for the endothelial dysfunction based on apoptosis, using a novel monkey model of aging, phylogenetically closer to humans but devoid of complications secondary to associated cardiovascular diseases [31]. They described an increased density of apoptotic cells observed in the endothelium of the aorta and femoral artery in old monkeys compared with young monkeys. 

### 3.4. Oxidative Stress and Inflammation in Aging-Related Diseases

As we have commented during the paper, aging is the major risk factor for the development of cardiovascular diseases and associated risk factors such as hypertension. In this context, vascular oxidative stress and inflammation significantly increase with age as a consequence of greater production of reactive oxygen species (ROS) and inflammatory markers. One of the main consequences of increased oxidative stress in aging is the functional inactivation of nitric oxide (NO) induced by elevated concentrations of superoxide anion, resulting in enhanced impairment of NO bioavailability and decreased vasodilator capacity [32, 33]. 

In the same line, inflammation is considered a critical initial step in the development of vascular disease during aging. In this regard, recent studies have demonstrated that arterial aging, in the absence of other known vascular risk factors, is associated with a proinflammatory profile [34, 35]. This proinflammatory state induces, among others, endothelial dysfunction by the upregulation of cellular adhesion molecules (VCAM-1 and ICAM-1), and this enhanced endothelial-leukocyte interactions and alterations in the secretion of different autocrine/paracrine factors which are pivotal in inflammatory response. In this sense, it is well known that the activation of transcriptional factors such as nuclear factor-kappa *β* (NF-*κ*B) are closely associated with these deleterious effects on vascular function [36]. In addition, NF-*κ*B activation increases during aging and is thought to be responsible of the increased expression of adhesion molecules and inducible nitric oxide synthase found in vascular wall [37]. TNF*α* signaling, mitochondrial ROS-induced pathways and local renin angiotensin system are known pathways that converge with NF-*κ*B and contribute to the vascular dysfunctionality observed during aging-related diseases.

Molecules strongly related to oxidative stress and aging are advanced glycation end products (AGEs) [38, 39]. Initially, AGEs were considered associated with hyperglycemic states. However, later studies demonstrated that AGEs formation may be stimulated even in normoglycemia although its expression is exaggerated under diabetes. AGEs are markers of carbonyl stress, which accumulates due to an increased level of sugars and reactive dicarbonyl compounds such as glucose, fructose, deoxyglucose glyoxal, and triosephosphates. AGEs, also termed glycotoxins, are well-known triggers of excess reactive oxygen species (ROS) and abnormally high oxidative stress [40, 41] that disrupt the structural integrity of proteins altering their interaction with other proteins and, therefore, affecting its functionality 

Large body of evidence has implicated AGEs as pathogenic mediators of the multiple complications associated with aging and cardiovascular diseases, such as arterial stiffness, myocardial relaxation abnormalities, atherosclerotic plaque formation, and endothelial dysfunction. In this sense, AGEs have a wide range of pathological effects, including increased vascular permeability, inhibition of vascular dilation by interfering with the nitric oxide (NO) pathway [42], LDL oxidation [43], and macrophage and endothelial cell activation to induce cytokine release and, thus, increase of oxidative stress [44]. In vitro studies have demonstrated quenching and inactivation of NO by AGEs, modulating NO activity and endothelium-dependent relaxation [45]. In addition, AGEs also induce the production of ROS, which favours the uncoupling of NO synthesis [46]. They also stimulate the synthesis and release of proinflammatory cytokines through the activation of NF-*κ*B [47, 48]. In experimental approaches, circulating levels of AGEs correlate with the level of different oxidative and inflammatory biomarkers, such as C reactive protein [49]. Upon such inflammatory situation, neutrophils, monocytes, and macrophages activate NADPH oxidase, leading to AGEs generation [50]. Also, AGEs have been shown to affect platelet adhesion and aggregation, thrombogenicity, and cell proliferation.

Recent findings have demonstrated that AGEs also are capable to augment hyperglycemia-associated depletion in endothelial nitric oxide production and endothelial nitric oxide synthase, demonstrating its important role in vascular dysfunction, linked to the induction of NO resistance [51]. This study observed an impaired vascular responsiveness to acetylcholine, accompanied by decreased eNOS protein expression and downregulation of cGMP-dependent protein kinase-1 expression.

Another deleterious mechanism on vascular function develop by AGEs is via the interaction with specific receptors called RAGEs. These receptors are members of the immunoglobulin superfamily that modulate the inflammatory response by increasing the expression of NF-*κ*B, proinflammatory cytokines, growth factors, and vascular adhesion molecules [52].

With respect to hypertension, it has been observed that AGEs diminish arterial compliance of large vessels elevating systolic blood pressure and pulse pressure, via inducing alterations in intima-media thickening and modifying collagen and elastic fibrils structure. Recent in vitro and in vivo studies have shown that angiotensin II type 1 receptor blockers can reduce AGEs formation [53, 54] although many doubts still exit about the concrete mechanism by which these molecules exerts this action. In summary, AGEs provide a new target for the development of more potent therapeutic agents in the treatment of vascular diseases.

In summary, the age-associated alterations in arterial structure and functionality, such as endothelial dysfunction and arterial stiffening, are considered as potent risk factors for arterial diseases, even after accounting for traditional cardiovascular risk factors, including arterial pressure.

## 4. New Molecular Mechanisms Associated with Hypertension and Aging

**Figure 2:**
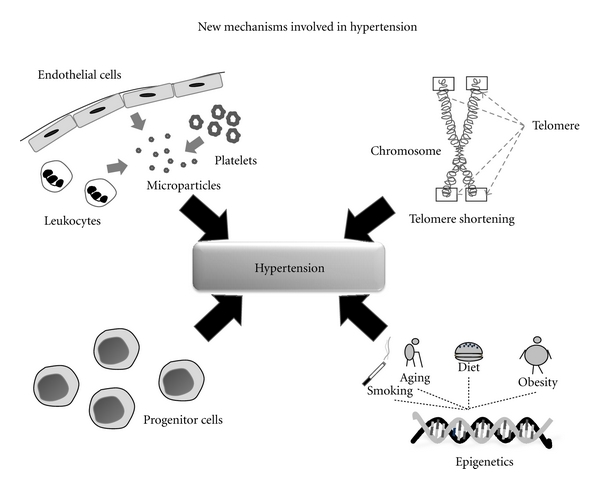
New mechanisms involved in hypertension in the elderly population. Telomere shortening, dysfunction of progenitor cells, increase of platelet, leukocyte, erythrocyte, and endothelial cells-derived microparticles, and epigenetic alterations together with the involvement of life style are proposed as new mechanisms associated with hypertension.

As we have described above, aging is a major risk factor for hypertension and cardiovascular disease. However, the molecular mechanisms by which increased blood pressure leads to vascular injury, and impaired endothelial function are not well defined. In recent years, new molecular mechanisms have appeared as possible targets to improve the knowledge of this pathology. The new mechanisms proposed include the effect of telomere shortening, dysfunction of progenitor cells, increase of deleterious microparticles, and finally epigenetics and the involvement of life style (Figure 2). 

### 4.1. Telomeres Length

In the last years, the “telomere hypothesis” of aging and its involvement in the development of aging-related diseases such as hypertension has gained importance [55]. Telomeres are distinctive DNA-protein structures at the end of linear chromosomes that preserve genomic integrity and progressively shorten with replication [56]. Telomeres have demonstrated age-dependent shortening in proliferative somatic cells [57], and accumulative evidence suggests that telomere length can be used as a marker of biological aging of the cardiovascular system and as a potential predictor of the risk and variability of developing hypertension and cardiovascular events. 

As telomere length becomes critically shortened, the cellular replicative machinery stops functioning and usually leads to replicative senescence. Studies in telomerase-deficient mice have shown a direct link between telomere shortening and hypertension [58]. In human studies, shorter telomere length has been strongly associated with oxidative stress [59], inflammation [60], atherosclerosis, and arterial stiffness [61]. Results of the Framingham Heart Study have also shown a reduced leukocyte telomere length in individuals with a higher renin-to-aldosterone ratio, especially in patients with hypertension [62]. In the same line, a five-year follow-up study has shown shorter leukocyte telomere length in hypertensive patients, who were also more likely to develop in the future atherosclerotic artery disease [63]. Moreover, this study presented telomere length as an independent risk factor for the development of cardiovascular disease both in patients with hypertension and in patients with normal blood pressure.

Interestingly, there are also diverse evidences showing that some of the drugs used in the treatment of hypertension stimulate telomerase activity and the reverse transcriptase that adds telomere repeats onto the ends of chromosomes, allowing the elongation of telomeres. The case of AT1 receptor antagonists of angiotensin II [64], statins [65], and aspirin [66] is becoming, therefore, a new target for future research studies.

During the last years, the number of studies about progenitor cells and its involvement in the development and evolution of vascular diseases has been increasing significatively. In this sense, the ability of endothelial progenitor cells to integrate into the vascular wall favouring endothelial repair has been widely described. Aging-related diseases are closely associated with endothelial injury and a significative reduction in the number and functionality of circulating EPCS. Studies in mouse models have demonstrated a crucial role of telomere shortening in the impairment of the progenitor cells during biological aging. In this regard, a recent study has shown that telomere shortening in EPCs plays an important role in the pathogenesis of cardiovascular disease via increased oxidative-related DNA damage [67].

In summary, telomere shortening could be a useful biomarker of biological aging of the cardiovascular system with a potent predictor value of the risk and variability of developing hypertension and associated cardiovascular events.

### 4.2. Progenitor Cells

As we have described previously, endothelial dysfunction is thought to be critical in the development of the vascular dysfunctionality observed in aging-related diseases such as hypertension and cardiovascular disease. Aging is related to the deleterious modifications observed in vascular function. In addition, impairments in endogenous vascular repair mechanisms, such as the conducted by EPCs, are thought to contribute to the manifestation of hypertension and cardiovascular disease. Bone marrow-derived EPCs have been considered as important agents of vascular repair. In addition, aging is associated with reduced number and function of EPCs [68], contributing to the greater cardiovascular risk observed in middle-aged and older adults. However, the real influence of aging on the increased EPCs apoptosis still remains unknown. Recently, Kushner et al. have documented that aging is associated with a proapoptotic EPC phenotype characterized by decreased expression of key antiapoptotic proteins associated with the Phosphoinositol-3-kinase signaling pathway and reduced telomerase activity, contributing to the diminished ability of EPCs to resist the apoptotic stimulus associated with aging [69].

It has been argued that the reduced endothelial repair capacity of EPCs in hypertensive patients is related to EPCs senescence and impaired endothelial function and likely represents an early event in the development of hypertension, contributing to the end-organ damage associated to this pathology [70, 71]. In this sense, infusions of circulating EPCs have been found to be able to augment endothelium-dependent vasodilatation, improving endothelial function [72]. Both, animals and human studies have demonstrated that the impaired EPC function that occurs during hypertension can be corrected with some antihypertensive treatments [73]. Therefore, it makes EPCs a modifiable factor that provides a new interesting line of research in the treatment of hypertension.

### 4.3. Circulating Microparticles

It was in 1967 when Wolf described the presence of small circulating procoagulant, prothrombotic, and proinflammatory particles in plasma called microparticles [74]. Circulating microparticles are small vesicular structures of about 0.1–1 *μ*m that have been associated with arterial thrombotic processes [75]. Microparticles are shed from the surface of different types of cells in response to activation, injury, and/or apoptosis. Although it is considered that the majority of circulating microparticles are platelet-derived (70%–90% of total), other cells such as red blood cells, leukocytes, and endothelial cells also release microparticles. 

Circulating microparticles are increased in patients with cardiovascular risk factors such as hypertension [76]. In this sense, increased number of circulating microparticles has been associated with poor clinical outcome [77] and, recently, microparticles have been defined as potential prognostic markers for vascular disease [78].

The influence of microparticles in vascular function, favouring endothelial dysfunction, has been demonstrated in multiple experimental studies. Microparticles have been shown to induce the expression of endothelial cyclooxygenase type 2, different adhesion molecules, the release of cytokines, and the impairment of nitric oxide release from vascular endothelial cells. Interestingly, these harmful effects on endothelial functionality seem to be mainly mediated by microparticles of endothelial origin although platelet-derived microparticles also mediate some of them. For this reason, most of the studies have focused on microparticles of endothelial origin as possible biomarkers of endotelial-dysfunction in patients with vascular disease. In this regard, when compared with classical markers of endothelial activation, endothelial derived microparticles appear to be more robust predictor of the incidence of coronary events [79]. For instance, it has been shown that circulating endothelial microparticles correlated positively with the extent and severity of coronary stenosis at angiography in patients with coronary syndromes [80].

It has been also observed that pharmacological treatment affects microparticle formation. While statins impair endothelial microparticle formation, aspirin seems not to affect microparticle formation, and others such as ticlopidine, abciximab, or cilostazol induced reduction in the number of circulating microparticles [81, 82]. Therefore, analysis of circulating microparticles could represent a useful tool in monitoring the efficacy of antihypertensive treatments.

Increasing evidence indicates that changes in plasma levels of microparticles of different cellular origins might be used as surrogate markers of vascular alterations, as those that occur during hypertension. However, there are still many questions of whether circulating levels of endothelial microparticles are the cause or result of endothelial dysfunction, and more studies will be needed. Thus, due to their procoagulant, prothrombotic, and proinflammatory effects on vasculature, microparticles could modulate the cross-talk between the cellular elements of the coagulative, thrombotic, and inflammatory systems through the transfer of different signaling molecules and receptors of their cellular origin to other cell types. Therefore, the study of circulating microparticles could be considered as novel therapeutic target in cardiovascular diseases. In this regard, novel proteomic approaches represents a new interesting tool for the study of the concrete microparticle composition, facilitating the identification of active components and clarifying their involvement in the development of diseases. In this context, the first proteomic studies analyzing the proteomic pattern of platelet [83], red blood cells [84], and endothelial cells-derived microparticles have already appeared [85].

### 4.4. Epigenetics and Lifestyle

Despite the considerable knowledge gained in recent years on the human genome, there are many questions regarding the mechanisms of inheritance and the mechanisms involved in hypertension and cardiovascular diseases. Nowadays, is fully accepted that the development of diseases is very directly involved with both genetic and epigenetic alterations.

In this regard, it is well known that genes are expressed or not depending on various factors such as chromatin and certain biochemical conditions, such as methylation of DNA and modification of histones. The science that studies all these factors is called epigenetics. It is important to realize that epigenetic processes are natural and essential for many body functions, but if they occur improperly they can cause serious adverse health effects, and hence the relevance of epigenetics in the study of human disease.

Epigenetics studies the interaction of DNA and its expression with the environment. It consists in the study of the inheritance of gene expression patterns that are not determined simply by the genetic sequence of each individual. It includes the study of any process that alters gene activity without changing DNA sequence and leads to changes that can be transmitted to daughter cells. We can say that epigenetics acts as “interlocutor” with the genetics and environment, helping to explain the action of lifestyle on the genes. It also describes the mechanisms that allow cells to respond quickly to environmental changes, thus providing a clear link between genes and the environment around them.

Hypertension is an arterial wall disease that together with smoking, high cholesterol, diabetes, overweight, and sedentary lifestyle is one of the main modifiable risk factor that leads to the development of cardiovascular diseases. Environmental factors such as diet, stress, and inactivity directly affect the incidence of this pathology, but it has also been established that stress in utero may program the later development of the disease. To date, the cardiovascular effects of early nutritional changes have been largely investigated following maternal undernutrition or protein restriction [86]. In this regard, interestingly, studies have shown that in maternal low-protein diet rat model, administration of angiotensin converting enzyme inhibitors or angiotensin receptor antagonists in early life can prevent the development of hypertension [87]. Epigenetic analysis demonstrated that the proximal promoter of the AT1b gene in the adrenal is significantly undermethylated. In vitro studies showed that the expression of this gene is highly dependent on promoter methylation, suggesting a link between fetal insults to epigenetic modification of genes and ultimately leading to the development of hypertension. Similarly, the methylation pattern of a serine protease inhibitor gene in human placenta is shown to be a marker for preeclampsia-associated hypertension.

The hallmark of hypertension in the elderly is increased vascular resistance. The usual therapeutic approach to the elderly hypertensive patient should generally consist of a reduction in salt and caloric intake and an increase in aerobic exercise. It has been demonstrated the effectiveness of modifications in the lifestyle to reduce blood pressure in aging patients. For instance, the Nonpharmacologic Interventions in the Elderly trial (TONE) demonstrated that with small reduction in salt or corporal weight, it is possible to reach a significantly reduction of blood pressure levels [88]. In this sense, epigenetics studies could provide new information about the effect of the environmental factors that are involve in the development of cardiovascular diseases. 

Nowadays, most of epigenetic studies are being conducted in the area of oncology, which are getting plenty of evidence linking epigenetic processes in cancer development. However, in the cardiovascular area, there is a great deal of untapped information. Therefore, epigenetics offer a promising avenue of investigation of the mechanisms involved in the development and evolution of cardiovascular diseases.

## 5. Summary

In the elderly population hypertension is a significant determinant of cardiovascular risk, and nowadays, it is well known that is closely associated with the incidence of vascular events. The development of vascular endothelial dysfunction is thought to be critical in the development of the vascular dysfunctionality observed in hypertension. In recent years, new molecular mechanisms such as telomere shortening, dysfunction of progenitor cells, increase of deleterious microparticles, and epigenetics alterations have appeared as possible targets to improve the knowledge of this pathology. Knowing more in depth the critical molecular mechanisms underlying the vascular dysfunctionality associated to hypertension may provide novel interventional treatments for promotion of cardiovascular health in older persons.
